# Inspirations on Virus Replication and Cell-to-Cell Movement from Studies Examining the Cytopathology Induced by *Lettuce infectious yellows virus* in Plant Cells

**DOI:** 10.3389/fpls.2017.01672

**Published:** 2017-09-27

**Authors:** Wenjie Qiao, Vicente Medina, Bryce W. Falk

**Affiliations:** ^1^Department of Plant Pathology, University of California, Davis, Davis, CA, United States; ^2^Department of Crop and Forest Sciences, University of Lleida, Lleida, Spain

**Keywords:** *Closteroviridae*, cytopathology, membrane remodeling, virus replication, plasmodesmata, intercellular movement

## Abstract

*Lettuce infectious yellows virus* (LIYV) is the type member of the genus *Crinivirus* in the family *Closteroviridae*. Like many other positive-strand RNA viruses, LIYV infections induce a number of cytopathic changes in plant cells, of which the two most characteristic are: *Beet yellows virus*-type inclusion bodies composed of vesicles derived from cytoplasmic membranes; and conical plasmalemma deposits (PLDs) located at the plasmalemma over plasmodesmata pit fields. The former are not only found in various closterovirus infections, but similar structures are known as ‘viral factories’ or viroplasms in cells infected with diverse types of animal and plant viruses. These are generally sites of virus replication, virion assembly and in some cases are involved in cell-to-cell transport. By contrast, PLDs induced by the LIYV-encoded P26 non-virion protein are not involved in replication but are speculated to have roles in virus intercellular movement. These deposits often harbor LIYV virions arranged to be perpendicular to the plasma membrane over plasmodesmata, and our recent studies show that P26 is required for LIYV systemic plant infection. The functional mechanism of how LIYV P26 facilitates intercellular movement remains unclear, however, research on other plant viruses provides some insights on the possible ways of viral intercellular movement through targeting and modifying plasmodesmata via interactions between plant cellular components and viral-encoded factors. In summary, beginning with LIYV, we review the studies that have uncovered the biological determinants giving rise to these cytopathological effects and their importance in viral replication, virion assembly and intercellular movement during the plant infection by closteroviruses, and compare these findings with those for other positive-strand RNA viruses.

## Introduction

Viruses are small obligate intracellular parasites that depend entirely on host cells for their replication. Viral replication and spread, accounting for the assembly and cell-to-cell movement of nascent virions, are fundamental events in the virus infection cycle that that determine successful viral infection in target hosts (Armas-Rillo et al., 2016). These processes occur in cellular compartments modified by specialized viral proteins that cause extensive membrane and organelle rearrangements in infected cells. The cellular remodeling during virus infection is best investigated with viruses that have a positive single-strand RNA genome (+ssRNA), which encompasses over one-third of all virus genera, including the majority of plant viruses (Ahlquist et al., 2003). Electron microscopy (EM) was used to observe the virus-induced cytopathic effects (i.e., cellular modifications) decades ago. With the development of novel modern techniques in molecular and cell biology, the observations obtained through EM have been reassessed and better interpreted nowadays, and the molecular mechanisms of the virus–host interactions that underlie the formation of virus-induced cellular remodeling are being addressed.

Viruses of the family *Closteroviridae* possess the largest and the most complex genomes (up to 20 kb) and virions (up to 2000 nm) of all +ssRNA plant-infecting viruses (Rubio et al., 2013), and cause phloem-limited infections in host plants. These features made viruses in the family *Closteroviridae* difficult to study. Although it has been realized that the virus-induced cytopathic changes in host cells often have various functions in viral replication, RNA translation and/or cell-to-cell transport (Miller and Krijnse-Locker, 2008; Laliberté and Sanfaçon, 2010; Jiang and Laliberté, 2016), however, relevant studies of closteroviruses have been more limited compared to those for other plant viruses. In this review, we provide an overview of the cellular modifications induced during closterovirus infection, especially those induced by *Lettuce infectious yellows virus* (LIYV), the type member of the genus *Crinivirus* in the family *Closteroviridae* (Wang et al., 2010). But the different types of well-studied cellular remodeling induced by other +ssRNA viruses, including how these alternations are formed and their roles in the virus infection cycle, are discussed, and we believe those may provide new understandings and perspectives on the molecular biology of closteroviruses and their interactions with host cells.

## The Family *Closteroviridae*

**FIGURE 1:**
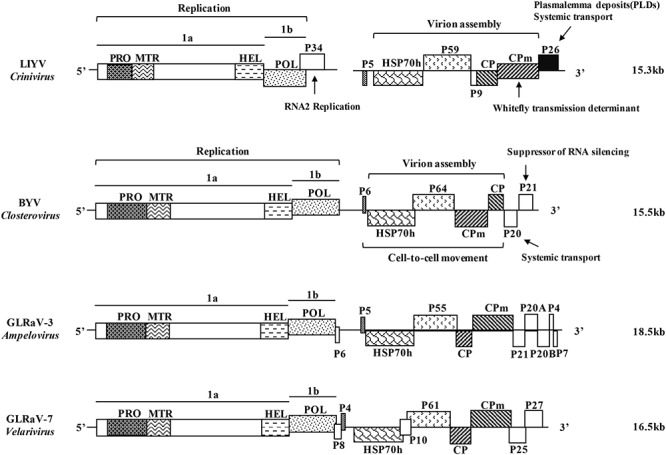
Schematic diagrams of the genome structure of the representative viruses in the four genera of the family *Closteroviridae*. LIYV, *Lettuce infectious yellows virus*, genus *Crinivirus*; BYV, *Beet yellows virus*, genus *Closterovirus*; GLRaV-3, *Grapevine leafroll-associated virus* 3, genus *Ampelovirus*; GLRaV-7, *Grapevine leafroll-associated virus* 7, genus *Velarivirus*. ORFs are shown as boxes, with the related domains indicated in the same in-fill. PRO, papain-like cysteine proteinase; MTR, methyltransferase; HEL, helicase; POL, RNA-dependent RNA polymerase; HSP70h, heat shock protein 70 homolog; CP, capsid protein; CPm, minor capsid protein; P, proteins named by their approximate molecular mass (e.g., P34, 34-kDa protein). The functional roles of some of the protein products are indicated with the well-studied LIYV and BYV. Genome size is labeled on the right side.

The family *Closteroviridae* comprises about 50 viruses^1^ that are segregated into four genera largely based on their genomic composition and vector species: *Closterovirus*, *Crinivirus*, and *Ampelovirus* are, respectively, transmitted by aphids, whiteflies, and mealybugs (Rubio et al., 2013; Agranovsky, 2016), while viruses of the genus *Velarivirus* represent a distinct monophyletic clade and lack of a known insect vector (Martelli et al., 2012; Melzer et al., 2013). Regardless of their mono- or bipartite genomes, all closteroviruses share two conserved gene modules that are involved primarily in replication and virion assembly, along with unique genes with no relationship found in other members of the family (**Figure 1**) (Dawson et al., 2013). The general molecular biology, genetic characteristics and evolution mechanisms of the family *Closteroviridae* were reviewed by (Dolja et al., 1994b, 2006; Agranovsky, 1996, 2016; Karasev, 2000). From the applied aspect, viruses in the family *Closteroviridae* cause severe diseases in various cultivated crops worldwide including citrus, beet, lettuce, tomato, cucurbits and grapevines, that lead to great economic losses. Their genetic similarity and threats on agriculture make the studies on the molecular biology of closteroviruses and their interactions with their plant host cells important. LIYV is the best studied crinivirus, its genome was sequenced and reverse genetics approaches were developed first (Klaassen et al., 1994, 1995, 1996). Studies on LIYV have proved to be critical in establishing a basic understanding of crinivirus–host interactions and their pathogenesis.

## Cytopathology of Closterovirus Infections

Since 1960, the cytopathological alterations induced by *Beet yellows virus* (BYV, genus *Closterovirus*) were studied in sugar beet and spinach (Esau, 1960). A similar pattern has been observed later with other closterovirus-infected plant cells: scattered and aggregates of virus particles of 12 nm in diameter, BYV-type inclusion bodies containing a fibrillar content, membrane proliferation, and/or degeneration and vesiculation of chloroplasts and mitochondria (Larsen et al., 1991; Faoro et al., 1992; Medina et al., 2003). The ultrastructural alterations of LIYV-infected tobacco (*Nicotiana benthamiana*, *N. clevelandii*), lettuce (*Lactuca sativa*) and goosefoot (*Chenopodium murale*) plants have also been described, and are mostly similar to those elicited by BYV and other closteroviruses (Hoefert et al., 1988; Pinto et al., 1988; Medina et al., 1998). Closterovirus cytopathology has been studied in different plant hosts (**Table 1**). In addition to those mentioned above, LIYV infections also induce a unique cytopathic effect: the electron-dense conical plasmalemma deposits (PLDs) (Medina et al., 1998).

**Table 1:** Summary of closteroviruses for which cytopathology has been studied in different host plants.

Virus	Genus	Host plants	Reference
*Beet pseudo-yellows virus* (BPYV)	*Crinivirus*	Lettuce (*Lactuca sativa*) Melon (*Cucumis melo*)	Medina et al., 2003
*Cucurbit yellow stunting disorder virus* (CYSDV)	*Crinivirus*	Cucumber (*Cucumis sativus*) Melon (*Cucumis melo*)	Medina et al., 2003
*Diodia vein chlorosis virus* (DVCV)	*Crinivirus*	Virginia buttonweed (*Diodia virginiana*)	Larsen et al., 1991
*Lettuce infectious yellows virus* (LIYV)	*Crinivirus*	Tobacco *(N. benthamiana, N. clevelandii*) Lettuce (*Lactuca sativa*) Goosefoot (*Chenopodium murale*)	Hoefert et al., 1988; Pinto et al., 1988; Medina et al., 1998, 2003
*Tomato infectious chlorosis virus* (TICV)	*Crinivirus*	Lettuce (*Lactuca sativa*)	Wisler et al., 1996; Medina et al., 2003
*Tomato chlorosis virus* (ToCV)	*Crinivirus*	Tomato (*Lycopersicon esculentum*) Tobacco (*N. clevelandii*)	Wisler et al., 1998; Medina et al., 2003
*Beet yellows virus* (BYV)	*Closterovirus*	Sugar beet (*Beta vulgaris* L.) New Zealand spinach (*Tetragonia expansa* Murr.) Goosefoot (*Chenopodium hybridum* L.)	Esau, 1960; Plaskitt and Bar-Joseph, 1978
*Carnation necrotic fleck virus* (CNFV)	*Closterovirus*	Carnation (*Dianthus caryophyllus*)	Bezić et al., 1984
*Citrus tristeza virus* (CTV)	*Closterovirus*	Sweet orange (*Citrus sinensis*) Mexican lime (*Citrus aurantifolia*)	Zhou et al., 2002
*Grapevine leafroll-associated virus* 2 (GLRaV-2)	*Closterovirus*	Tobacco (*N. benthamiana*) Grapevine (*Vitis vinifera*)	Castellano et al., 2000
*Grapevine leafroll-associated virus* 1 (GLRaV-1)	*Ampelovirus*	Grapevine (cv. Merlot)	Faoro and Carzaniga, 1995
*Grapevine leafroll-associated virus* 3 (GLRaV-3)	*Ampelovirus*	Grapevine (cvs Barbera, Cannonau, Croatian, Merlot and Moscato)	Faoro et al., 1992; Faoro and Carzaniga, 1995; Maree et al., 2013
*Grapevine leafroll-associated virus* 7 (GLRaV-7)	*Velarivirus*	Grapevine (*Vitis vinifera*)	Castellano et al., 2000

### BYV-Type Inclusion Bodies

Closterovirus infections are mostly limited to phloem-associated cells, although cytopathic effects of some closteroviruses can also be found occasionally in the mesophyll and epidermal cells (Esau and Hoefert, 1971; Bar-Joseph et al., 1977). The most characteristic inclusion bodies of closterovirus infections are represented by ∼100 nm double-membrane vesicles (DMVs) and multivesicular complexes (MVCs; bunches of single-membrane vesicles surrounded by a common membrane), and often have associated virus particles (Agranovsky, 2016). The membranes of DMVs and MVCs are likely derived from cell membranes of endoplasmic reticulum (ER) in the case of BYV (Plaskitt and Bar-Joseph, 1978), LIYV (Hoefert et al., 1988) and *Grapevine leafroll-associated virus*-2 (GLRaV-2, genus *Closterovirus*) (Castellano et al., 2000), or mitochondria in the case of GLRaV-1 and GLRaV-3 (genus *Ampelovirus*) (Faoro et al., 1992; Faoro and Carzaniga, 1995). These vesiculated membranous inclusion bodies have been speculated to be associated with closterovirus replication, but evidence so far is mostly based on comparisons to what is known for other viruses, and from studies based on predicted protein functions.

Two lines of evidence directly support that closterovirus DMVs and MVCs are associated with replication. The genomes of viruses of the family *Closteroviridae* are characterized by the 5′-terminal replicative module consisting of ORFs 1a and 1b which code for the conserved domains of papain-like cysteine proteinase (PRO), methyltransferase (MTR), helicase (HEL) and RNA-dependent RNA polymerase (POL) (**Figure 1**). For LIYV this gene module is contained in RNA1. Inoculation of tobacco protoplasts with *in vitro* transcripts of only LIYV RNA1, or an RNA1 mutant coding only the ORFs 1a and 1b proved to be sufficient for efficient replication and concomitant formation of the DMVs and MVCs (Medina et al., 1998; Wang et al., 2010). Since LIYV encodes structural proteins on RNA2, these data suggest that the DMVs or MVCs are associated with LIYV replication. Immunogold labeling of BYV-infected tissue indicated the co-localization of BYV replication-associated proteins (PRO, MTR, HEL) with the membranous vesicle aggregates, supporting the role of these cellular structures as replication platforms (Erokhina et al., 2001; Zinovkin et al., 2003). Gushchin et al. (2013) also found a hydrophobic segment of the putative membrane-binding domains in the BYV ORF1a central region (CR) can reorganize the perinuclear ER and form uniform globules ∼1 μm in diameter, which were speculated to be involved in the formation of the membranous closterovirus DMVs and MVCs. A conserved domain of the CR, called “the Zemlya region,” has been found in all members of the genus *Closterovirus* through sequence analysis, might have similar roles on cellular membrane modification during virus infection (Gushchin et al., 2017).

### Plasmalemma Deposits (PLDs)

**FIGURE 2:**
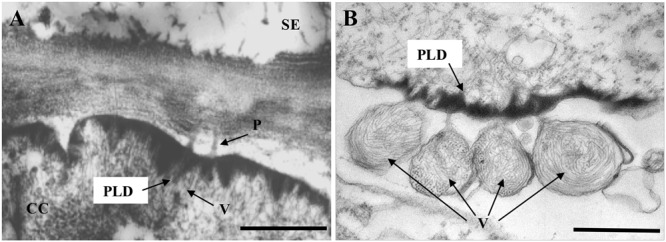
Transmission electron micrographs showing LIYV-induced conical plasmalemma deposits (PLDs). **(A)** Shows PLDs located at the internal side in a companion cell (CC) of a LIYV-infected *Nicotiana benthamiana* leaf, associated with LIYV virions (V) and plasmodesmata (P) [image is modified from Medina et al. (2003) with permission of John Wiley and Sons]. **(B)** Shows PLDs in a LIYV-infected *N. benthamiana* protoplast, sacks of LIYV virions (V) are external to the plasmalemma directly adjacent to abundant PLDs [image is modified from Kiss et al. (2013) under the CC BY License]. Labeling is CC, companion cell; P, plasmodesmata; PLD, plasmalemma deposit; SE, sieve element; V, LIYV virions.

Accumulation of electron-dense material at the plasmalemma (PM) has been observed with infections of several closteroviruses such as *Citrus tristeza virus* (CTV) (Zhou et al., 2002), *Tomato chlorosis virus* (ToCV) and *Beet pseudo-yellows virus* (BPYV) (Medina et al., 2003), but so far only LIYV infections are known to induce characteristic conical electron-dense PLDs (**Figure 2**). PLDs were first described by Hoefert et al. (1988) and Pinto et al. (1988) and have been found in all LIYV-infected host plants examined so far. These conical crystalline-like structures are found located at the internal side of PM in companion cells (CC) and phloem parenchyma, just over plasmodesmata (PD) pit fields between these cells or adjoining sieve elements (SE). LIYV virions are consistently observed through transmission electron microscopy (TEM) to be associated with the PLDs, and appear to be oriented perpendicular to the PM and PD between cells (Pinto et al., 1988; Medina et al., 2005; **Figure 2A**). Virus-like particles were also observed within PD under the PLDs extending from a phloem parenchyma cell into the adjacent sieve element (Pinto et al., 1988). The PLDs have also been observed in LIYV-infected protoplasts with virus particles arranged perpendicular to the PM, and sacks of LIYV particles were found external to the PM adjacent to the PLDs (Kiss et al., 2013; **Figure 2B**). LIYV virions located near the PLDs were confirmed by immunogold labeling with antibodies against LIYV structural proteins (HSP70h, P59, CP, and CPm) (Medina et al., 2005). All this evidence leads to speculation for PLDs having roles in virus movement, possibly by orienting virus particles near the PD and/or aiding the shuttling between phloem parenchyma and CC, and/or into SE for systemic transport (Stewart et al., 2009).

In searching for the LIYV determinant(s) of the PLDs, antibodies to the LIYV RNA 2-encoded non-virion protein P26 showed specific labeling of the PLDs in LIYV infected plants and protoplasts (Medina et al., 2005). Furthermore, P26 expressed from the heterologous TMV (*Tobacco mosaic virus*) vector was shown to be sufficient to induce the formation of the PLDs indistinguishable from those produced by LIYV infection, however, unlike LIYV particles, TMV particles were not observed associated with the PLDs (Medina et al., 2005; Stewart et al., 2009), which might indicate specific interactions between LIYV virions and the P26-aggregated PLDs. It is worth mentioning that although all described criniviruses have an ORF at the 3′ terminus of RNA2 encoding a similar-sized protein, the PLD formation has been reported only for LIYV P26. Whether they have roles in virus infection cycle similar to that of the LIYV-encoded P26 is yet unknown. Mutational analyses using LIYV *in vitro* transcripts showed that in-frame stop codons introduced into LIYV P26 ORF (P26X) did not affect LIYV RNA accumulation in tobacco protoplasts (Yeh et al., 2000). Agroinoculation of LIYV for systemic infection of *N. benthamiana* plants was developed by Wang et al. (2009), and our recent results have confirmed that P26X has no effects on viral replication and virion formation, but results in disrupted systemic infection. The hypothesis is P26 might be interacting with LIYV virion components and/or some host factors to direct LIYV virions to the cell periphery, and/or to facilitate the intercellular movement of virus particles through the PD.

With all the above efforts, progress has been made to reveal the connections between closterovirus-induced cellular remodeling and their roles in virus replication and movement, but definitive documentation is still limited. Several fundamental questions still remain unsolved, such as closterovirus and host factors involved in the formation of the cellular structures, content of these structures and the role of each individual component in virus infection. Positive strand RNA viruses share some common features in the structure and function of utilizing cellular membranes for virus replication, and in the case of plant viruses modifying PD for intercellular movement. Closterovirus-induced BYV-type inclusion bodies are found similar to structures known as ‘viral factories’ in cells infected with diverse types of animal and plant viruses, generally as sites for virus replication. LIYV-induced PLDs, although so far unique to LIYV infection, the fundamental mechanisms of targeting and modifying PD may be analogous to those for other +ssRNA viruses. Therefore, in the following sections, different types of membrane and plasmodesmata modification strategies induced by +ssRNA viruses for their replication and movement will be discussed, which may shed light on further studies of closteroviruses.

## Virus-Induced Membrane Modification

Positive-strand RNA viruses are known to induce membrane and organelle rearrangements in the cytoplasm of infected cells. Despite the great diversity of viruses from different families, the membrane alterations generally involve the formation of spherules and vesicles derived from a variety of organelles, including the ER, mitochondria, peroxisomes, lysosomes and chloroplasts (Denison, 2008; Laliberté and Sanfaçon, 2010; Harak and Lohmann, 2015). Those host-derived membranes serve as scaffolds for the assembly of ‘viral factories,’ which provide a platform for anchoring the viral replication complexes (VRCs) and create a protected environment for RNA synthesis and viral genome encapsidation. Some virus factories have been shown to associate with and traffic along microfilaments, and may be involved in other processes such as viral RNA (vRNA) translation and cell-to-cell virus transport (Armas-Rillo et al., 2016; Jiang and Laliberté, 2016). The closterovirus DMVs and MVCs appear similar to the vesicles found for other plant and animal virus infections, such as those for *Turnip mosaic virus* (TuMV), *Potato virus X* (PVX), *Hepatitis C virus* (HCV) and *Poliovirus* (PV) (Romero-Brey and Bartenschlager, 2014; Jiang and Laliberté, 2016). The formation and functional mechanisms of the vesiculated closterovirus inclusion bodies are not get understood. Inspired by the observations and speculations from studies on closterovirus-induced cytopathic effects, below we describe some well-studied examples of membrane modifications induced by +ssRNA viruses.

### Vesicle Type

**FIGURE 3:**
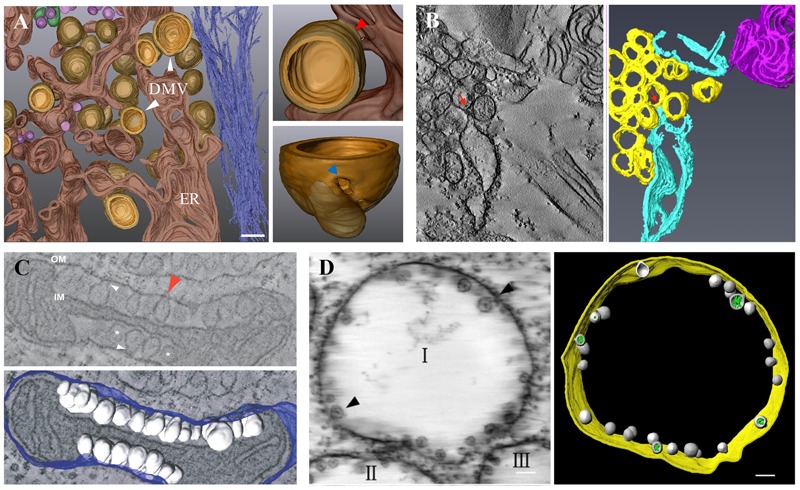
Virus-induced membrane modification. **(A)** 3D architecture of HCV-induced membrane rearrangements. HCV DMVs are protrusions from the ER membrane into the cytosol. The enlarged DMV structure displays a connection between the outer membrane of a DMV and the ER membrane (red arrow) and a pore-like opening that connects the interior of the DMV with the cytosol (blue arrow). **(B)** ET and 3D reconstruction of TuMV-induced membrane rearrangement. SMVs (yellow) are in close proximity or connected (red arrows) to dilated rER (sky blue). **(C)** ET and 3D reconstruction of FHV-induced spherule rearrangements of a mitochondrion. Numerous spherules (white) are shown as outer mitochondrial membrane (OM, blue) invaginations with interiors connected to the cytoplasm by a necked structure (arrows). IM, inner mitochondrial membrane. **(D)** ET and 3D model of BBSV-induced membrane rearrangements. Spherules (gray) are shown within ER-derived vesicle packets (indicated by I, II, III) and are connected to the ER outer membrane (gold) through neck-like structures (arrows). Green, fibrillar materials inside the spherules. **(A)** is adapted from Romero-Brey et al. (2012) under the CC BY License; **(B)** is adapted from Wan et al. (2015a) with permission of the American Society for Microbiology; **(C)** is adapted from Kopek et al. (2007) under the CC BY License; **(D)** is adapted from Cao et al. (2015) with permission of the American Society for Microbiology.

The morphotype exemplified by the closterovirus DMVs involves the formation of membranes with positive curvature (exvaginations of the membrane of various organelles). This is also the predominant characteristic of the replication factories of hepaci-, corona-, arteri-, and picornaviruses (Romero-Brey and Bartenschlager, 2014). Those are often shown to be motile and morphologically dynamic. HCV is a member of the genus *Hepacivirus* in the family of *Flaviviridae*, a major cause of liver diseases in humans. The most prominent membranous structures in HCV-infected cells are DMVs, ∼150 nm in diameter, the kinetics of their appearance correlates with kinetics of vRNA replication (Romero-Brey et al., 2012; Ferraris et al., 2013). Electron tomography (ET) and three-dimensional (3D) reconstructions identified HCV DMVs as ER membrane protrusions into the cytosol, with ∼45% of DMVs the outer membrane is connected to the ER membrane bilayer via a short neck-like structure, which might represent an intermediate stage of DMV formation before released from the ER (Romero-Brey et al., 2012; **Figure 3A**). Most of the DMVs appeared as closed structures, only ∼8% were found having an opening (‘pore’) toward the cytosol (Romero-Brey et al., 2012). Whether this opening corresponds to “immature” DMVs before closure or represents a distinct structure remains to be elucidated. The presence of double-stranded RNA (dsRNA) and enzymatically active viral replicase with purified DMVs suggests these vesicles are the sites of vRNA synthesis (Ferraris et al., 2010; Paul et al., 2013). Whether RNA replication takes place within DMVs or on their outer surface remains unclear. The sole expression of HCV NS5A has shown to be sufficient to induce DMV formation, but with a low efficiency, the concerted action of other non-structural viral proteins NS3, NS4A, NS4B, and NS5B is still required for the formation of the membranous webs (MWs), that are composed of vesicles embedded in a membrane matrix (Romero-Brey et al., 2015). In addition to viral proteins, cellular factors involved in this membrane remodeling were identified, e.g., cyclophilin A (CypA) and phosphatidylinositol 4-kinase IIIα (PI4KIIIα) that are associated with NS5A, regulating the formation and integrity of DMVs (Chatel-Chaix and Bartenschlager, 2014). Furthermore, the HCV vesicles are also involved in viral intracellular transport, the small replication factories show fast, saltatory movement that is microtubule (MT)-dependent, a direct interaction between NS3/NS5A and tubulin/actin has been studied (Lai et al., 2008; Wölk et al., 2008).

Another prominent example of the ER-derived vesicles are those induced by TuMV, a member of the genus *Potyvirus* in the family *Potyviridae*. Ultrastructural characterization of TuMV-induced cellular rearrangements reveals the formation of rough ER (rER) connected convoluted membranes early in infection, followed by the production of single membrane vesicle (SMV) and DMV-like structures, which are later shown to be tubules by ET generated 3D-model (Grangeon et al., 2012; Wan et al., 2015a; **Figure 3B**). The viral membrane protein 6K2 induces the formation of these cytoplasmic vesicle-like structures, similar to its orthologs encoded by other potyviruses like *Tobacco etch virus* (TEV) (Beauchemin et al., 2007). Those vesicle-like structures contain vRNA and several replication-related viral and host proteins, including viral POL, VPg and the host translation eukaryotic initiation factor (iso) 4E [eIF(iso)4E], heat shock cognate 70-3 (Hsc70-3), poly(A)-binding protein (PABP), and the eukaryotic elongation factor 1A (eEF1A), are thus referred to as sites for TuMV replication (Beauchemin and Laliberté, 2007; Beauchemin et al., 2007; Dufresne et al., 2008; Thivierge et al., 2008). Similar to HCV, TuMV-induced vesicles are mobile, but instead of MT, microfilaments as well as the secretory pathway and myosin motor proteins are required for the intracellular and intercellular movement of the vRNA (Cotton et al., 2009; Agbeci et al., 2013; Jiang et al., 2015). They are also found present in phloem SE and xylem vessels as a form for long-distance movement (Wan et al., 2015b). Other plant viruses that induce ER-derived vesicles include *Cowpea mosaic virus* (CPMV), *Grapevine fanleaf virus* (GFLV), PVX, *Tomato mosaic virus* (ToMV), etc (Jiang and Laliberté, 2016).

### Spherule/Invagination Type

Some viruses like noda-, bromo-, toga- and flaviviruses induce the formation of spherules or vacuoles toward the lumen of the targeted cell organelle by membrane invaginations in infected cells. These appear to be static in opposition to the motile, ER-derived vesicles (Romero-Brey and Bartenschlager, 2014; Jiang and Laliberté, 2016). *Flock house virus* (FHV), the best studied member of the family *Nodaviridae*, infects insect cells but also replicates and assembles infectious progeny after transfection into mammalian, plant, *Saccharomyces cerevisiae*, and *Caenorhabditis elegans* cells (Den Boon and Ahlquist, 2010). FHV is the first virus whose replication complexes were visualized three-dimensionally in detail by ET, numerous spherules of 50–70 nm in diameter are shown as outer mitochondrial membrane invaginations with interiors connected to the cytoplasm by a necked structure of ∼10 nm in diameter (Kopek et al., 2007; **Figure 3C**). FHV RNA replication intermediates, nascent RNA and replication protein A were detected within membrane-bound spherules by immunofluorescence microscopy, thus validating the spherules as FHV-induced compartments for vRNA synthesis (Kopek et al., 2007; Short et al., 2016). Protein A was found to be necessary, but not sufficient for spherule formation, the presence of a replication-competent RNA template is also required. Without RNA template, protein A induces only clustering and zippering of the mitochondrial surfaces (Kopek et al., 2010).

*Beet black scorch virus* (BBSV) is a member of the genus *Necrovirus* in the family *Tombusviridae* that causes severe yield loss of sugar beet production. BBSV infection induces the invagination of the ER membranes, leading to the formation of 50–70 nm spherules within ER-derived vesicle packets (Cao et al., 2015). ET analysis and 3D architecture studies reveal that these packets are distinctively connected to each other via a tubule-like structure with a diameter of 15–30 nm, spherules are connected to the ER outer membrane through neck-like structures, indicating a connection between the interiors of spherules and the surrounding cytoplasm (Cao et al., 2015; **Figure 3D**), similar in ultrastructural appearance to *Brome mosaic virus* (BMV)-induced membrane structures (Schwartz et al., 2002; Laliberté and Zheng, 2014). BBSV dsRNAs and the auxiliary replication protein P23 were found localized within viral spherules, indicating them as the sites for VRC assembly and BBSV replication. The expression of BBSV P23 protein alone alters ER structure in ways resembling that in BBSV infection, although not sufficient to be called spherules (Cao et al., 2015).

As discussed above, two main architectures of remodeled membranes have been proposed, the vesicle type and the spherule/invagination type. The well-studied and representative +ssRNA viruses that infect different hosts from different kingdoms and induce the typical membrane morphotypes are selected as examples, which reveal that, albeit with their own distinct traits, similarities are present among vesicle morphology, formation and roles in virus infections, of related or even unrelated viruses, suggesting viruses might adopt conserved strategies during long-term evolution to utilize host cellular membranes to accomplish their infection cycle. Viral non-structural (NS) proteins primarily contribute to these membrane and organelle alterations that often have specificity in recognizing organelles and contain hydrophobic domains acting on membrane targeting and rearrangement. For example, FHV protein A is a transmembrane protein with an N-proximal mitochondrial localization signal and hydrophobic transmembrane domain (Miller and Ahlquist, 2002), while HCV NS5A, TuMV 6K2 and BBSV P23 are all ER-localized membrane proteins (Penin et al., 2004; Cao et al., 2015; Jiang et al., 2015). Host factors are absolutely essential to form and regulate these membranous structures, some have been identified but are still limited, a summary of the host factors contributing to replication complex biogenesis can be found in Harak and Lohmann (2015).

## Virus-Induced Plasmodesmata Modification

Unlike animal viruses, plant viruses use plasmodesmata (PD) for cell-to-cell and systemic infection. They are plasma membrane-lined channels as symplasmic connections between adjacent cells, composed of appressed ER in the center (the desmotubule, DT) that are often tethered tightly (at most 10 nm in diameter) to the PM by unidentified spokes (Benitez-Alfonso et al., 2010; Nicolas et al., 2017). The space between DT and PM, referred to as the cytoplasmic sleeve (CS), places a limit on the size of materials that can be transported, and is defined as the size exclusion limit (SEL). This allows for the passive diffusion of small molecules but restricts the intercellular trafficking of macromolecular structures such as virions and non-encapsidated viral ribonucleoprotein complexes (vRNPs). Virus-encoded movement proteins (MPs) are required for viral cell-to-cell movement through PD. These can interact with other viral or host factors to target and modify PD by two main characterized mechanisms: ‘tubule-guided’ movement, which involves the extensive modification of PD into MP-lined tubules that mostly results in disappearance of DT and overall dilated PD pores [e.g., GFLV, *Broad bean wilt virus* (BBWV2) (Xie et al., 2016)]; ‘non-tubule-guided’ movement, in which viruses move through PD as virions or vRNPs by regulating the PD SEL and likely relying on the cellular machinery that transports macromolecules without inducing major PD structural changes (e.g., TMV, PVX, TuMV, BYV) (Heinlein, 2015b; Kumar et al., 2015; Rojas et al., 2016).

### ‘Tubule-Guided’ Movement

Tubule-guided movement was found to be common among positive-strand RNA viruses including como-, nepo-, olea-, and trichoviruses, as well as some ambisense ssRNA (e.g., tospoviruses) and dsDNA (e.g., caulimoviruses) viruses (Rojas et al., 2016). GFLV is a member of the genus *Nepovirus*, family *Secoviridae*, GFLV MPs are able to assemble a tubular transport structure inside modified PD that allows the intercellular transport of virus particles. Expression of GFLV MP forms tubules that protrude from the PM of transfected protoplasts, similar to that observed with BBWV 2 and CPMV (Van Lent et al., 1991; Liu et al., 2011). So far the MPs of tubule-forming viruses have been known to interact with the CP of the respective virus, indicating the movement specificity and a possible mechanism of the tubule structures transporting viral particles (Belin et al., 1999; Carvalho et al., 2003). Tubule formation by the GFLV MP depends on a functional secretory pathway. The use of biochemical inhibitors was shown to disturb tubule formation and MP localization (Kumar et al., 2015). Consistent with these findings, GFLV MP was found to interact with a PD-localized, receptor-like protein (PDLP1) to mediate tubule assembly and virus movement. PDLP1 is transported to PD via the ER-Golgi secretory pathway in a myosin-dependent manner, which may facilitate the assembly of MP into tubules by acting as a catalyst or providing a PD docking platform (Amari et al., 2011). The microtubular cytoskeleton also seems to be involved in the transport of MP to the plasma membrane and the cell periphery, its inhibition results in MP accumulation in the cytoplasm (Laporte et al., 2003). Furthermore, modification of the cell wall by callose deposition and cellulose reduction was observed on PD containing BBWV2 VP37-tubules via immunegold labeling and 3D ET construction (Xie et al., 2016). Callose at the neck region of the PD plays an important role in the regulation of PD permeability, however, whether those modifications are associated with the formation or function of VP37-derived tubules are still unknown (De Storme and Geelen, 2014; Tilsner et al., 2016).

### ‘Non-tubule-guided’ Movement

Viruses that do not form MP tubules for intercellular movement can be further divided into two groups based on their movement format as vRNPs (e.g., tobamo- and potexviruses) or virions (e.g., poty- and closteroviruses). The prototype viruses exemplifying the vRNP type of movement are TMV (CP-independent) and PVX (CP-dependent). TMV MP has shown properties that facilitate the cell-to-cell movement mainly as binding the ssRNA, localization to the ER and PD and a capability to increase the PD SEL (Citovsky et al., 1990; Liu and Nelson, 2013; Yuan et al., 2016). The vRNPs of TMV have shown to be associated with VRCs by the TMV MP-vRNA binding, i.e., moving cell-to-cell in a form of MP-VRC, which are targeted to the PM and PD using cytoskeletal network (Kawakami et al., 2004; Amari et al., 2014). Host proteins that impact TMV spreading through interactions with TMV MP have been described in detail (Liu and Nelson, 2013).

Unlike tobamoviruses, the movement of potexviruses depends on the CP in addition to three MPs encoded in overlapping ORFs referred to as the ‘triple gene block’ (TGB) (Verchot-Lubicz et al., 2010). TGB1 is shown to possess the capacity to bind ssRNA, increase PD SEL and facilitate the cell-to-cell movement of vRNA by interacting with TGB2/3 and CP (Howard et al., 2004; Samuels et al., 2007). TGB2/3 are ER transmembrane proteins, that induce ER-derived granular vesicles moving along the ER network via the actin cytoskeleton and myosins (Ju et al., 2005; Bamunusinghe et al., 2009). They act as accessory factors delivering the TGB1-vRNA-CP complex to and through PD, while themselves do not move through PD (Haupt et al., 2005; Kumar et al., 2015).

In contrast, potyviruses and closteroviruses move through PD possibly in the form of virions (perhaps modified in some way) and have no dedicated MP(s) but use several viral proteins that have additional roles in virus multiplication (Heinlein, 2015b; Rojas et al., 2016). The cylindrical inclusion protein (CI), CP, helper-component proteinase (HC-Pro), viral genome-linked protein (VPg) and P3N-PIPO have been indicated involved in cell-to-cell movement of potyviruses. CI is an RNA helicase essential for virus movement via the formation of conical deposits at the cell periphery adjacent to PD that may function in aligning and passing the filamentous virions to and through PD (Rodríguez-Cerezo et al., 1997; Roberts et al., 1998; Zilian and Maiss, 2011; Revers and García, 2015). PD localization of CI is modulated by P3N-PIPO through trafficking along the ER-Golgi pathway and interacting with PM localized host protein (Wei et al., 2010; Vijayapalani et al., 2012). CP and HC-Pro have shown to mediate the intercellular movement by modifying PD SEL, moving cell-to-cell, and facilitating vRNA movement (Rojas et al., 1997). CP is also detected, often in linear arrays, near the vertices or inside PD-localized CI cones and in PD (Rodríguez-Cerezo et al., 1997). VPg, HC-Pro and CI are detected at protruding tips at the 5′ end (presumably) of virus particles of *Potato virus A* (PVA), suggesting a model for intercellular movement in which these tips act as a guide device through the interaction between CI and HC-Pro/VPg for directional trafficking of the modified virion complexes to and through PD (Puustinen et al., 2002; Torrance et al., 2006; Gabrenaite-Verkhovskaya et al., 2008; Revers and García, 2015). Virion assembly and cell-to-cell movement of TEV are abolished by mutations in the conserved core region of CP, also indicating some potyviruses likely move as virions (Dolja et al., 1994a). On the other hand, TuMV 6K2-induced vesicles are found move intracellularly via actin microfilaments and intercellularly through PD (Grangeon et al., 2013; Jiang et al., 2015). The presence of 6K2 vesicles containing vRNA and the viral RdRp in phloem SE and in xylem vessels are also detected by immunohistolocalization (Wan et al., 2015b). This evidence leads to another hypothesis that TuMV and potentially other potyviruses move cell-to-cell as replication-competent complexes containing viral CP and RNA. Whether potyviruses move in the form of encapsidated virions, or CP-genome-containing 6K2 complexes, or both remains to be tested further (Heinlein, 2015a).

The polar structure of virus particles observed with PVA is similar to that of closteroviruses, and has been proposed to represent a general mechanism for directional trafficking and cotranslational disassembly of these filamentous viruses (Taliansky et al., 2008; Benitez-Alfonso et al., 2010). Closteroviruses possess exceptionally long (650–2000 nm), filamentous virus particles with a unique bipolar morphology: the major CP encapsidates most of the genomic RNA, a minor CP (CPm) incorporated with other viral-encoded proteins coats a short 5′-terminal fragment. BYV virions are known to be comprised of five viral proteins: CP, CPm, HSP70h, P64 and P20 (Peremyslov et al., 2004a). Genetic analysis showed that CP, CPm, P64, and HSP70h are all essential for BYV cell-to-cell movement, suggesting the ‘tailed’ virion structure as a prerequisite for intercellular movement (Alzhanova et al., 2000, 2001). BYV HSP70h has been shown to localize to PD autonomously in a myosin VIII dependent manner and may serve as a ‘driving force’ for targeting the virion to PD, while the translocation of the virion through PD may be powered by the ATPase activity of HSP70h (Avisar et al., 2008). In addition, BYV cell-to-cell movement is shown to require another non-structural ER-localized protein P6 (Peremyslov et al., 2004b). P20 is dispensable for intercellular transport but is necessary for systemic movement (Prokhnevsky et al., 2002).

In the case of LIYV, mutational analysis has shown that the structural proteins CP, Hsp70h and P59 are required for LIYV systemic infection, however, whether they act as factors for cell-to-cell movement like those of BYV, or for systemic movement remains unclear (unpublished). Surprisingly, CPm is dispensable for systemic infection but is required for whitefly transmission (Stewart et al., 2010). The subcellular localization of CP, HSP70h and P59 was tested by transiently expressed GFP-fusion protein, but none showed PD localization like BYV HSP70h (unpublished). As mentioned above, LIYV P26 forms the unique conical electron-dense PLDs that are often associated with PD and virus particles, morphologically resembling the conical deposits formed by potyvirus CI. LIYV P26X delivered by *Agrobacterium tumefaciens* is shown not able to infect plants systemically, its roles in cell-to-cell movement of the virion complex are yet unknown. It would be interesting to identify the viral and/or host factors for the intracellular movement of P26 targeting to PD and the molecular connections among the PLDs structure, virions and the PD channels.

Furthermore, it needs to be mentioned that to carry out cell-to-cell and systemic movement, plant viruses also need to pass through the phloem tissues via the successive crossings of the bundle sheath (BS), vascular parenchyma cells (VP), and CC to SE, where viruses can move with the phloem translocation stream to distant sink organs, which is more notable for the phloem-limited closterovirus infections (Hipper et al., 2013). PD connections between different plant cells are not necessarily equivalent, e.g., specialized PD are found connecting CC-SE that exhibit a larger SEL and are thus more permissive than the PD between mesophyll cells. Moreover, several studies have shown that virus transport can be specifically impeded at certain borders, indicating distinct PD permeability and precise regulation at these boundaries (Wang et al., 1998; Hipper et al., 2013). Whether the proposed P26 or other LIYV-encoded proteins are involved in these processes remains unclear, while how the transported or vector delivered closteroviruses exit form SE to CC and VP to initiate new infection sites is another question waiting to be answered.

In summary, a diversity of mechanisms and strategies utilized by different types of plant viruses for cell-to-cell movement are briefly introduced above. We have mainly focused on the viral factors that target and regulate the PD structure for their intercellular movement. Viral proteins and host factors involved in the intracellular transport (through the host cytoskeleton and secretory pathway) and the phloem transport of plant viruses were reviewed in detail previously (Boyko et al., 2000; Nelson and Citovsky, 2005; Harries et al., 2010; Schoelz et al., 2011; Hipper et al., 2013).

## Conclusion and Future Perspectives

Positive-strand RNA viruses encode membrane-associated viral proteins to actively modify cellular membranes to assemble viral factories as functional sites for virus replication, translation, and/or assembly. Plant viruses cause systemic infections of their hosts by intracellular movement targeting to PD along host cytoskeleton and membrane systems, cell-to-cell movement by passing through PD and long-distance movement through phloem vasculature following the source-to-sink transportation. These biological processes required for virus infection cycles can be directly reflected as cytopathic effects in infected cells and visualized through TEM and other technologies.

*Lettuce infectious yellows virus* infection causes two characteristic cellular changes: the BYV-type inclusion bodies and the PLDs that are suspected to be related to LIYV replication and cell-to-cell movement, respectively. The molecular connections between cellular modifications and closterovirus infection are intriguing but need further efforts. For our interests in deciphering the molecular mechanisms of these not yet well-defined structures, in this article, we provide an overview of the representative types of membrane remodeling and PD modifications induced by +ssRNA viruses from different families, which may provide important insights and information for the relative studies on closteroviruses. Some well-studied viruses whose cellular changes have shown high morphological similarities to those of closteroviruses are exemplified, and may indicate similar biogenesis and functional mechanisms. Our knowledge of the cellular ultrastructure induced by positive-strand RNA viruses has increased substantially but is still limited. However, the rapid development of genetic manipulation and cell biology techniques and advanced imaging system such as the 3D ET will contribute to reveal details of membrane morphologies and identify the viral and host factors involved in their biogenesis and underlying functional mechanisms.

## Statement

All appropriate permissions have been obtained from the copyright holders of the figures that have been reproduced in the article. **Figure 2A** is modified from Medina et al. (2003) with permission of John Wiley and Sons; **Figures 3B,D** are adapted, respectively, from Wan et al. (2015a) and Cao et al. (2015) with permissions of the American Society for Microbiology; **Figures 2B**, **3A,C** are adapted from Kiss et al. (2013), Romero-Brey et al. (2012) and Kopek et al. (2007) under the terms of the Creative Commons Attribution (CC BY) License.

## Author Contributions

WQ drafted the manuscript, VM and BF gave critical revision of the article.

## Conflict of Interest Statement

The authors declare that the research was conducted in the absence of any commercial or financial relationships that could be construed as a potential conflict of interest.

## Footnotes

1 http://ictvonline.org/virusTaxonomy.asp
